# Supplementary material for: RUFY3 links Arl8b and JIP4-Dynein complex to regulate lysosome size and positioning
Source: Nat Commun. 2022 Mar 21;13:1540. doi: 10.1038/s41467-022-29077-y (PMC8938454; doi:10.1038/s41467-022-29077-y)
Supplement: Supplementary file 1 — Supplementary Information [file 41467_2022_29077_MOESM1_ESM.pdf]

Supplementary Information

RUFY3 links Arl8b and JIP4-Dynein complex to regulate lysosome size and positioning

Gaurav Kumar[1], Prateek Chawla[2], Neha Dhiman[2], Sanya Chadha[1], Sheetal Sharma[1], Kanupriya Sethi[1], Mahak Sharma[2] and Amit Tuli[1*]

[1]Divison of Cell Biology and Immunology, CSIR-Institute of Microbial Technology (IMTECH), Chandigarh (India)
[2]Department of Biological Sciences, Indian Institute of Science Education and Research (IISER)-Mohali, Punjab (India)

[*]Corresponding author

Email: atuli@imtech.res.in

Running title: RUFY3 regulates size and positioning of lysosomes

Supplementary Fig. 1

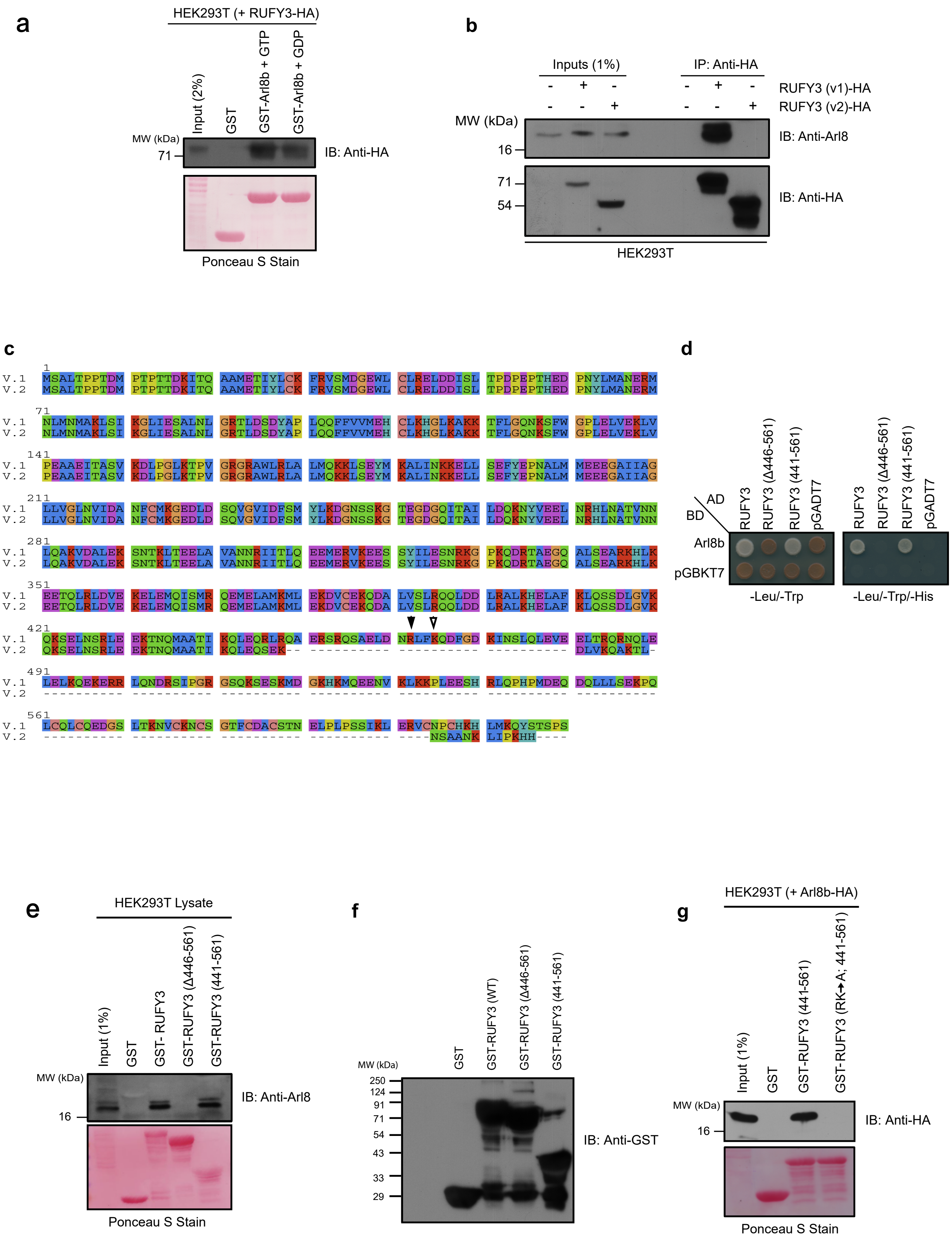

Supplementary Fig. 1: Variant 1 but not variant 2 of RUFY3 specifically interact with Arl8b. a GST-pulldown assay. Recombinant GST and GST-Arl8b (bound to GDP or GTP) proteins were immobilized on glutathione-conjugated-agarose beads and incubated with HEK293T cell lysate expressing RUFY3-HA. The precipitates were IB with anti-HA antibodies and GST-tagged proteins were visualized using Ponceau S staining. **b** HA-tagged-RUFY3 variant 1 (620 aa) or variant 2 (469 aa) expressing plasmid was transfected into HEK293T cells. The lysates were IP with anti-HA antibodies-conjugated-agarose beads and the precipitates were IB with the indicated antibodies. **c** Schematic showing protein sequence alignment of RUFY3 variant 1 with variant 2. The arrowheads mark basic/positively charged residues (R462 and K465) present in RUFY3 variant 1 that are crucial for binding with Arl8b. **d** Yeast two-hybrid assay. Plasmids encoding GAL4-AD fused to RUFY3 (WT), RUFY3 (Δ446-561) and RUFY3 (441-561) were co-transformed with Arl8b (WT) fused to GAL4-BD in *Saccharomyces cerevisiae* to examine the interactions. The co-transformants were spotted on non-selective medium (-Leu/-Trp) to confirm viability and on selective medium (-Leu/-Trp/-His) to detect interactions. **e** HEK293T cell lysates were incubated with recombinant GST or indicated GST-RUFY3 fusion proteins bound to glutathione resin. Precipitates were analyzed by Western blotting with anti-Arl8 antibodies. GST-tagged proteins were visualized using Ponceau S staining. **f** Immunoblot of recombinant GST-tagged RUFY3 proteins used in **Fig. 1f.** The presence of GST, GST-RUFY3 (WT), GST-RUFY3 (Δ446-561 aa) and GST-RUFY3 (441-561 aa) was confirmed by immunoblotting using anti-GST antibody. **g** Recombinant GST or indicated GST-RUFY3 fusion proteins bound to glutathione resin were incubated with lysates from HEK293T cells expressing Arl8b-HA. The precipitates were immunoblotted with anti-HA antibodies and GST-tagged proteins were visualized using Ponceau S staining.

Supplementary Fig. 2

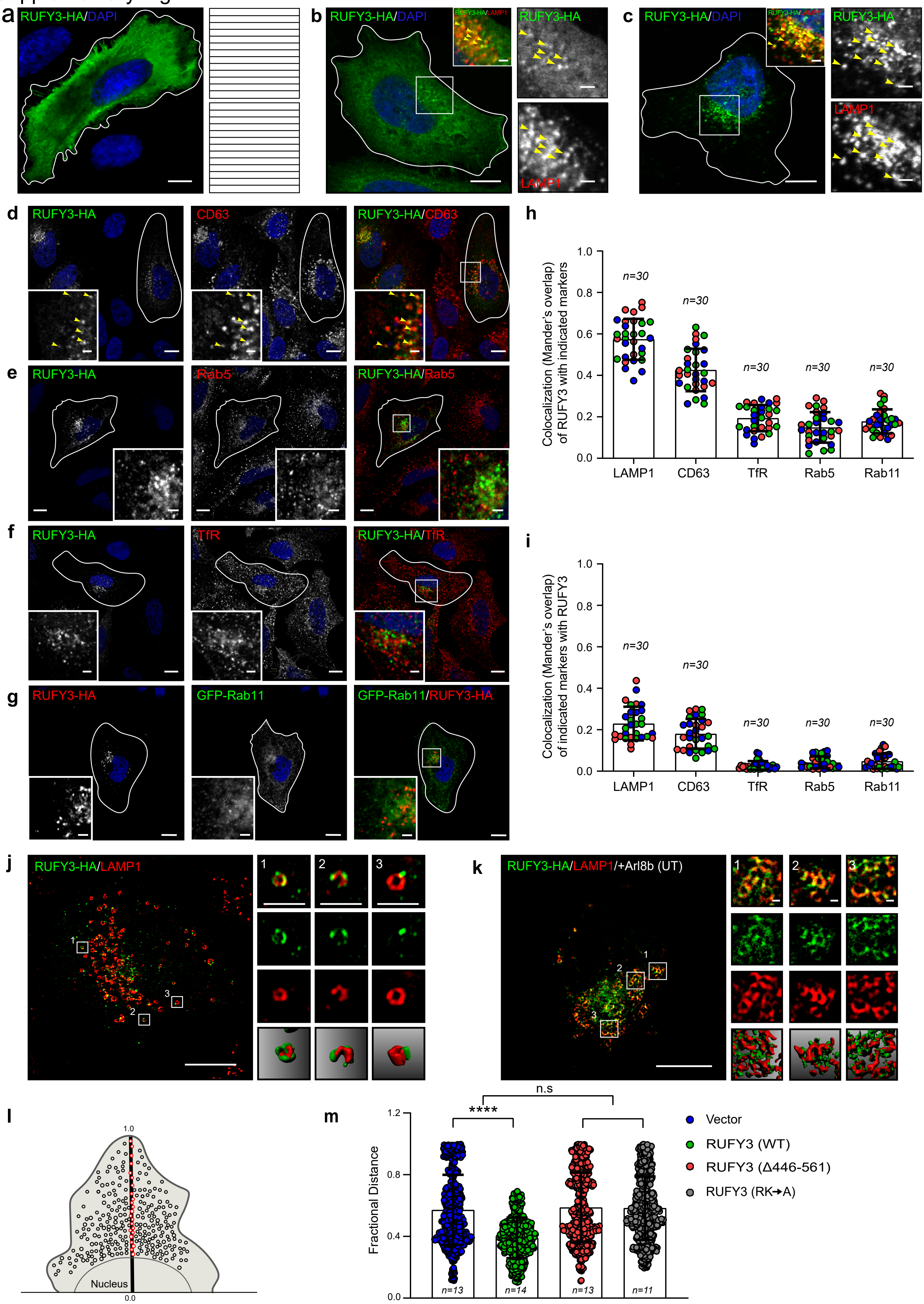

Supplementary Fig. 2: RUFY3 localizes to CD63 and LAMP1 positive compartments. a-c Representative confocal micrographs of HeLa cells transfected with RUFY3-HA plasmid showing varying level of RUFY3 expression. Post fixation, cells were stained for lysosomes and RUFY3 using anti-LAMP1 and anti-HA antibodies, respectively. The transfected cells are outlined and in the insets, a magnified region of the boxed area is shown, depicting the localization of RUFY3 on LAMP1-positive compartments as indicated by yellow arrowheads. Note, in **c**, cells were briefly permeabilized with 0.05% saponin for 5 min on ice in order to remove cytosol pool of RUFY3 followed by fixation step. Scale Bars: 10 µm (main); 2 µm (inset). **d-g** Representative confocal micrographs of HeLa cells transfected with RUFY3-HA expressing construct and stained for different endocytic markers. A magnified region of the boxed area is shown in the insets depicting colocalized pixels of RUFY3-HA with different marker proteins. Scale Bars: 10 µm (main); 2 µm (inset). **h, i** Colocalization of RUFY3-HA with indicated markers was measured using Mander's overlap. Values plotted are mean ± S.D. from three independent experiments. Experiments are color-coded, and the total number of cells analyzed is indicated on the graph. **j, k** Representative SIM image of a HeLa cell transfected with RUFY3-HA or co-transfected with Arl8b (untagged) and stained with indicated antibodies. Insets in **j** show magnification of selected vesicles, highlighting the presence of RUFY3 on the LAMP1-positive vesicles. The fourth row of insets shows an isosurface view of vesicles generated using Imaris software. The insets in **k** depict enhanced colocalization of RUFY3 on lysosomes in the presence of Arl8b. Scale Bars: 10 µm (main); 2 µm (inset). **l** Schematic illustrating the methodology for calculating fraction distance of lysosomes from center of the nucleus. **m** The graph represents quantification of fraction distance of lysosomes for experiments performed in **Fig. 2a-c**. Values plotted are mean ± S.D. and the total number of cells analyzed is indicated on the graph (*****p*<0.0001; n.s., not significant; two-tailed Student's *t*-test).

Supplementary Fig. 3

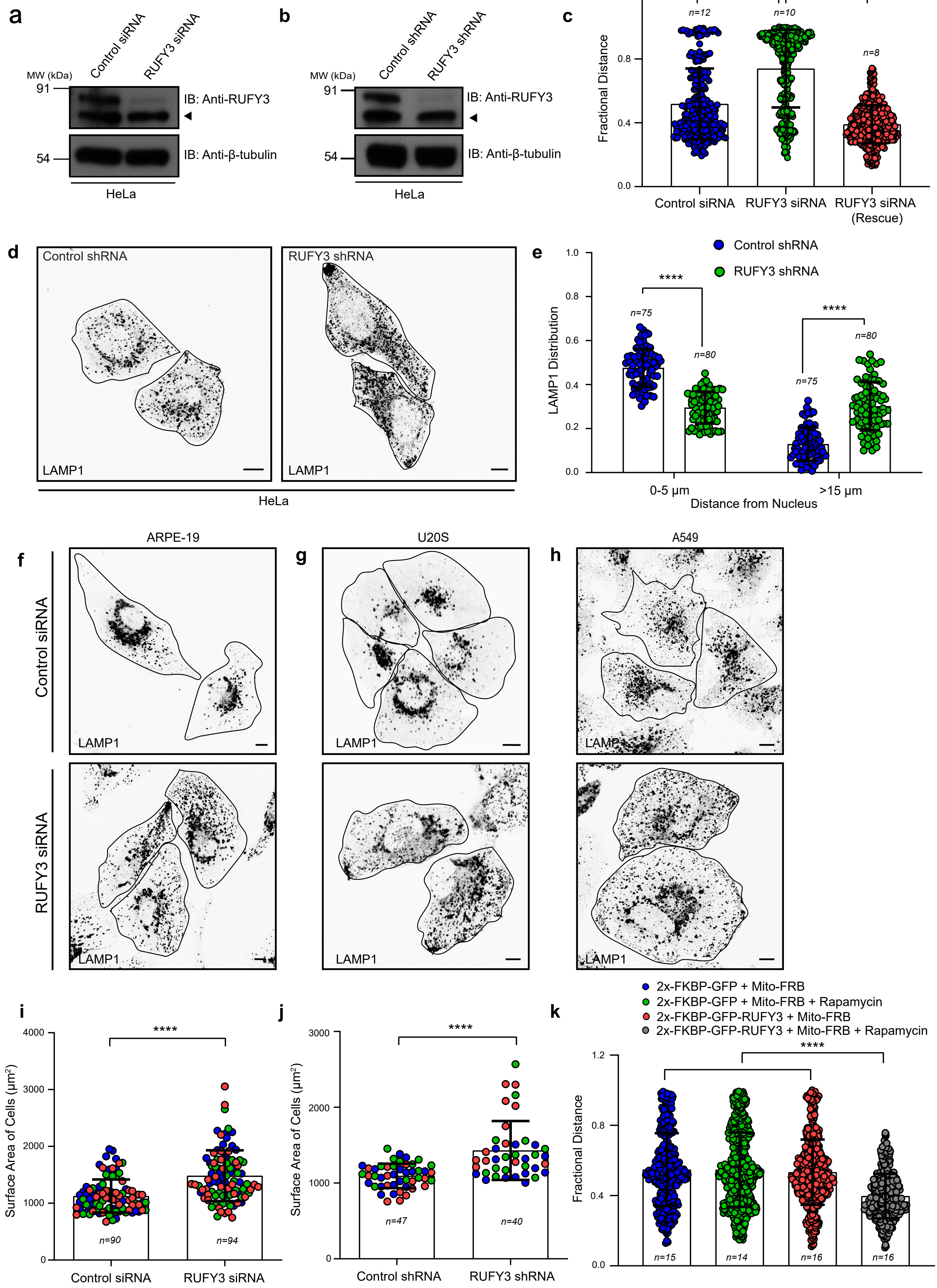

Supplementary Fig. 3: Silencing of RUFY3 expression leads to the peripheral distribution of lysosomes in different cell types. a, b Immunoblot showing RUFY3 knockdown efficiency in control and RUFY3 depleted HeLa cells using siRNA and shRNA treatment. The arrowhead corresponds to a non-specific signal detected by the anti-RUFY3 antibody. The blot was also probed for β-tubulin for the equal amount of protein loading. **c** The graph represents quantification of fraction distance of lysosomes for experiments performed in **Fig. 3a**. Values plotted are mean ± S.D. and the total number of cells analyzed is indicated on the graph (****p<0.0001; two-tailed Student's *t*-test). **d** Representative confocal micrographs (represented as grayscale inverted) showing lysosome distribution in HeLa cells expressing control shRNA or RUFY3 shRNA. The lysosomes were stained using an anti-LAMP1 antibody. Scale Bar: 10 µm. **e** The distribution of LAMP1-positive lysosomes was quantified from the experiments presented in **d**. Values plotted are mean ± S.D. from three independent experiments. The total number of cells analyzed is indicated on the graph (****p<0.0001; two-tailed Student's *t*-test). **f-h** Representative confocal microscopy images (represented as grayscale inverted) of control and RUFY3-silenced ARPE-19 (f), U20S (**g**), A549 (**h**) cells stained with an anti-LAMP1 antibody. Scale Bars: 10 µm. **i, j** Quantification of the surface area of HeLa cells transfected with either control siRNA or RUFY3 siRNA (**i**) and control shRNA or RUFY3 shRNA (**j**). Values plotted are mean ± S.D. from three independent experiments. Experiments are color-coded and the total number of cells analyzed is indicated on the graph (****p<0.0001; two-tailed Student's *t*-test). **k** The graph represents quantification of fraction distance of mitochondria for experiments performed in **Fig. 3h**. Values plotted are mean ± S.D. and the total number of cells analyzed is indicated on the graph (****p<0.0001; two-tailed Student's *t*-test).

Supplementary Fig. 4

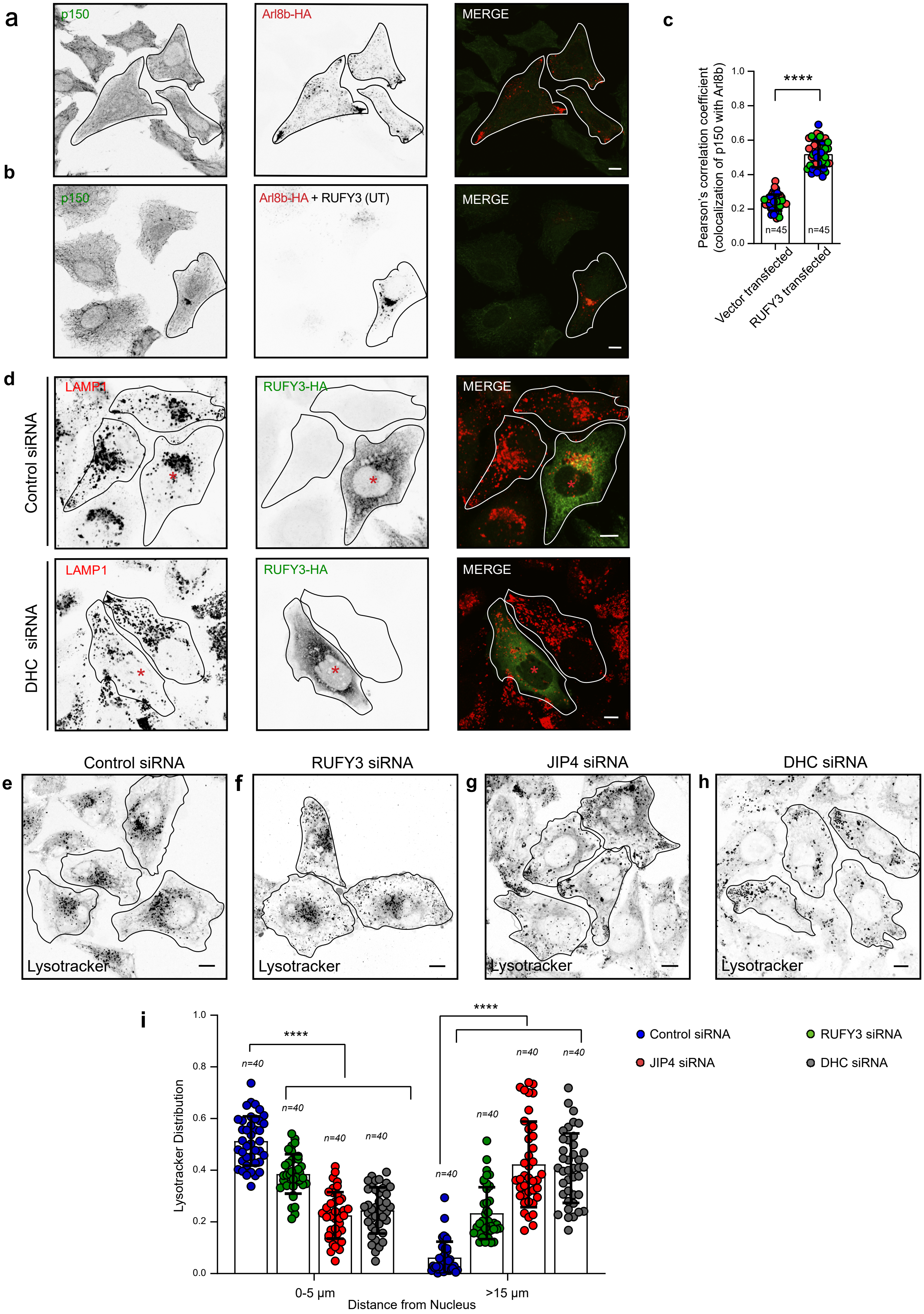

Supplementary Fig. 4: RUFY3 links Arl8b and JIP4 and mediates lysosome positioning in a dynein-dependent manner. a-c Representative confocal microscopy images (represented as grayscale inverted) of HeLa cells transfected with Arl8b-HA (**a**) or co-transfected with Arl8b-HA and RUFY3 (UT). Cells were fixed and stained with anti-p150 and anti-HA antibodies and transfected cells are outlined. Scale Bars: 10 µm. Colocalization of p150 with Arl8b from these experiments was quantified using Pearson's correlation coefficient. Values plotted are mean ± S.D. from three independent experiments. Experiments are color-coded and the total number of cells analyzed is indicated on the graph (****p<0.0001; two-tailed Student's t-test). **d** Representative confocal microscopy images of HeLa cells treated with control or dynein heavy chain (DHC) siRNA and transfected with plasmid expressing RUFY3-HA. Cells were fixed and stained with anti-LAMP1 and anti-HA antibodies. The asterisk marks transfected cells. Scale Bars: 10 µm. **e-i** Representative confocal microscopy images (represented as grayscale inverted) of HeLa cells transfected with indicated siRNAs and stained for lysosomes using Lysotracker. Scale Bars: 10 µm. The quantification of distribution of Lysotracker-labeled lysosomes in HeLa cell transfected with indicated siRNA is shown in **i** and values plotted are mean ± S.D. from three independent experiments. The total number of cells analyzed is indicated on the graph (****p<0.0001; two-tailed Student's t-test).

Supplementary Fig. 5

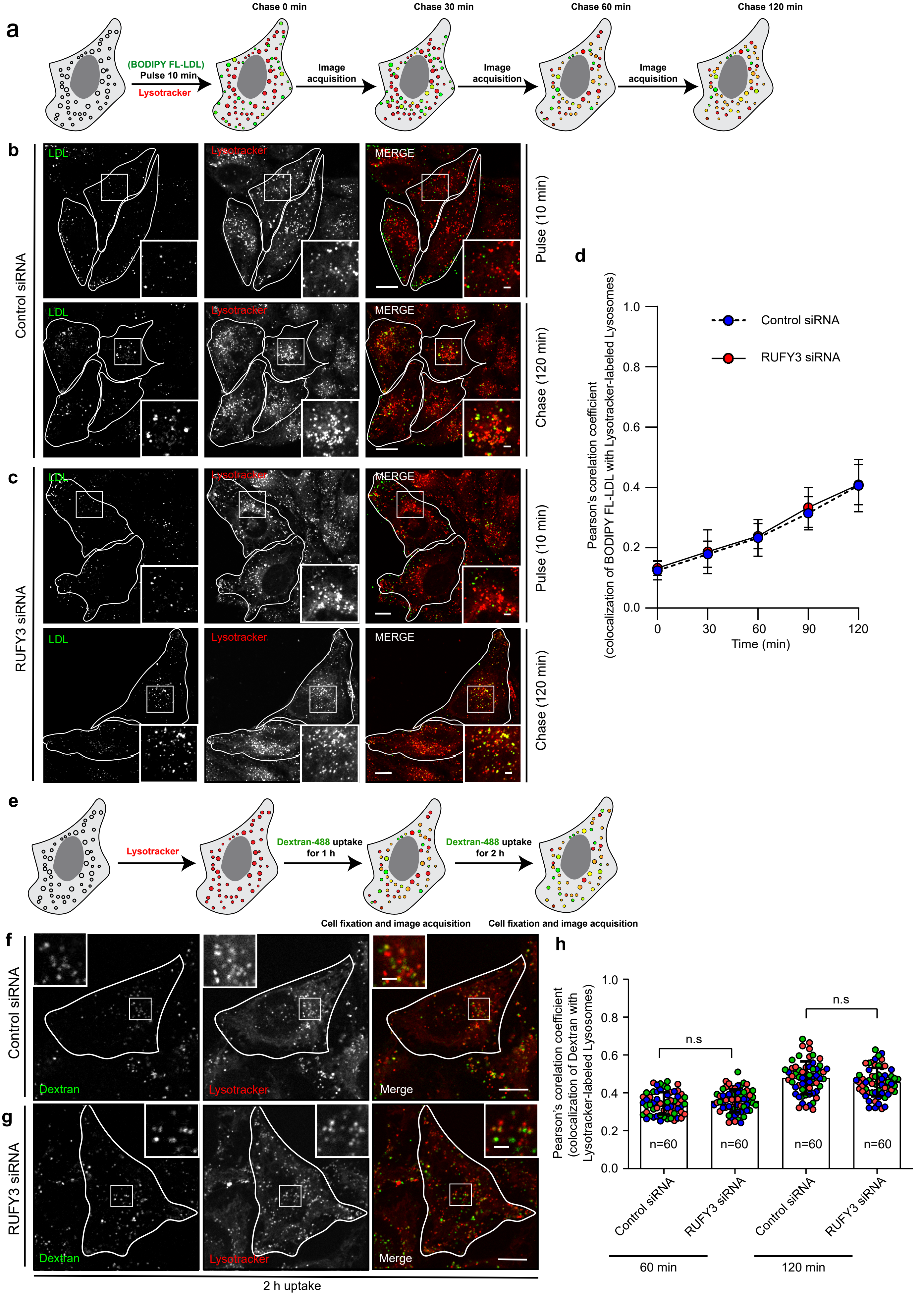

Supplementary Fig. 5: RUFY3 depletion does not affect endocytic cargo trafficking to lysosomes. a Schematic represents the methodology used to the trafficking of LDL to lysosomes. **b-d** Representative confocal micrographs of BODIPY FL-LDL trafficking to Lysotracker-labeled lysosomes in control and RUFY3-silenced HeLa cells for the indicated time periods. Insets show a magnified view of boxed areas highlighting delivery of BODIPY FL-LDL to Lysotracker-labeled lysosomes. Scale Bars: 10 µm (main); 2 µm (inset). Pearson's correlation coefficient was quantified for the images and values plotted are mean ± S.D. from three independent experiments. **e** Schematic represents the methodology used to study the dextran delivery to lysosomes. **f-h** Representative confocal micrographs of dextran delivery to Lysotracker-labeled lysosomes in control and RUFY3-silenced HeLa cells for the indicated time periods. Insets show a magnified view of boxed areas highlighting delivery of pulsed dextran to Lysotracker-labeled lysosomes. Scale Bars: 10 µm (main); 2 µm (inset). Pearson's correlation coefficient was quantified for the images and values plotted are mean ± S.D. from three independent experiments. Experiments are color-coded and the total number of cells analyzed is indicated on the graph (n.s., not significant; two-tailed Student's *t*-test).

Supplementary Fig. 6

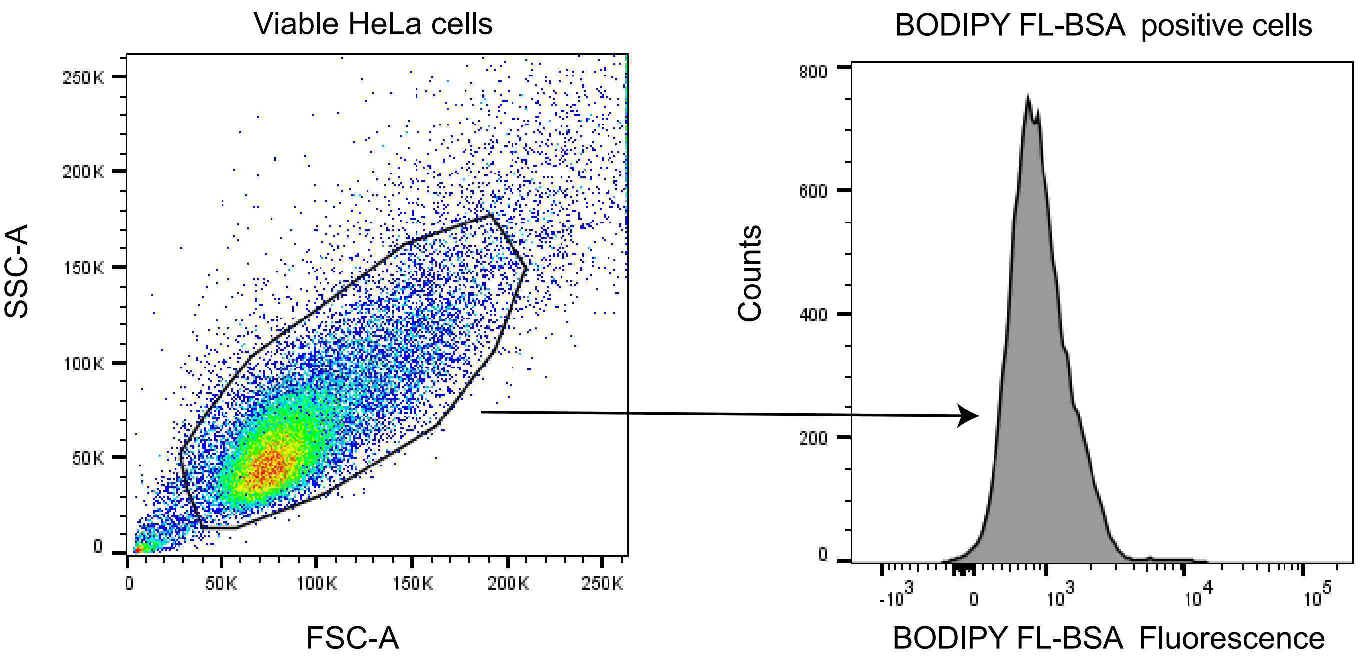

Supplementary Fig. 6: Gating strategy for the measurement of BODIPY FL-BSA uptake in HeLa cells by Flow cytometry. Example of gating strategy used to measure BODIPY FL-BSA fluorescence signal in HeLa cells. All Flow cytometry analysis were performed on 30,000 cells and viable cells were first gated on a plot of SSC-A versus FSC-A. The data panel presented here corresponds to one of the nine biological replicates of HeLa cells treated with control siRNA and pulsed with BODIPY FL-BSA for 2 h (**Fig. 8k-m**). Similar gating strategy was used for the experiments shown in **Fig. 7c, d**.

Supplementary Table 1: List of DNA constructs used in this study.

Plasmid Name	Description	Source
***Yeast two-hybrid constructs*:**		
pGADT7	GAL4-activation domain yeast two-hybrid vector	Clontech
pGADT7-RUFY3 (WT)	Full-length human RUFY3 (1-620 aa) cloned into the pGADT7 vector	This study
pGADT7-RUFY3 (Δ446-561)	Human RUFY3 lacking amino acids 446-561 cloned into the pGADT7 vector	This study
pGADT7-RUFY3 (441-561)	Human RUFY3 amino acids 441-561 cloned into the pGADT7 vector	This study
pGADT7-RUFY3 (RK→A)	Human RUFY3 with point mutations at amino acid positions R462 and K465 to A; cloned into the pGADT7 vector	This study
pGAD-C1-RILP	RILP cloned into the pGAD-C1 vector	Gift from Prof. Mitsunori Fukuda
pGBKT7	GAL4-DNA binding domain yeast two-hybrid vector	Clontech
pGBKT7-Arl8b (WT)	Human Arl8b (lacking first 17 aa) cloned into the pGBKT7 vector	Described previously (Marwaha et al., 2017)
pGBKT7-Arl8b (Q75L)	Human Arl8b (lacking first 17 aa) with Q75L point mutation cloned into the pGBKT7 vector	Described previously (Marwaha et al., 2017)
pGBKT7-Arl8b (T34N)	Human Arl8b (lacking first 17 aa) with T34N point mutation cloned into the pGBKT7 vector	Described previously (Marwaha et al., 2017)
pGBD-C1-Rab7	Human Rab7a cloned into the pGBD-C1 vector	Described previously (Marwaha et al., 2017)
Mammalian expression constructs:		
pcDNA3.1(-)	Mammalian expression vector	Invitrogen
pcDNA3.1(-)-RUFY3 (UT)	Full-length human RUFY3 (1-620 aa) without any tag cloned into the pcDNA3.1(-) vector	This study

pcDNA3.1(-)- RUFY3-FLAG	C-terminal FLAG-tagged full-length human RUFY3 (1-620 aa) cloned into the pcDNA3.1(-) vector	This study
pcDNA3.1(-)-RUFY3 (RK→A)-FLAG	C-terminal FLAG-tagged human RUFY3 with point mutations at amino acid positions R462 and K465 to A; cloned into the pcDNA3.1(-) vector	This study
pcDNA3.1(-)- RUFY3-FLAG (RESCUE)	C-terminal FLAG-tagged full-length human RUFY3 (1-620 aa) rescue construct against RUFY3 siRNA cloned into the pcDNA3.1(-) vector	This study
pcDNA3.1(-)- RUFY3-HA	C-terminal HA-tagged full-length human RUFY3 (1-620 aa) cloned into the pcDNA3.1(-) vector	This study
pcDNA3.1(-)-RUFY3 (Δ446-561)-HA	C-terminal HA-tagged human RUFY3 lacking amino acids 446-561; cloned into the pcDNA3.1(-) vector	This study
pcDNA3.1(-)-RUFY3 (RK→A)-HA	C-terminal HA-tagged human RUFY3 with point mutations at amino acid positions R462 and K465 to A; cloned into the pcDNA3.1(-) vector	This study
pcDNA3.1(-)- RUFY3 (v2)-HA	C-terminal HA-tagged human RUFY3 (1-469 aa; variant 2) cloned into the pcDNA3.1(-) vector	This study
pEGFP-C1	EGFP expressing mammalian expression vector	Clontech
pEGFP-C1-RUFY3 (WT)	Full-length human RUFY3 (1-620 aa) cloned into the pEGFP-C1 vector	This study
pEGFP-C1-RUFY3 (RESCUE)	Full-length human RUFY3 (1-620 aa) rescue construct against RUFY3 siRNA cloned into the pEGFP-C1 vector	This study
pcDNA3.1(+)-Arl8b (UT)	Full-length human Arl8b (untagged; UT) cloned into the pcDNA3.1(+) vector	Described previously (Marwaha et al., 2017)
pcDNA3.1(-)-Arl8b (WT)-FLAG	Full-length human Arl8b with C-terminal FLAG tagged cloned into the pcDNA3.1(-) vector	This study
pcDNA3.1(-)-Arl8b (WT)-HA	Full-length human Arl8b with C-terminal HA tagged cloned into the pcDNA3.1(-) vector	Described previously (Marwaha et al., 2017)

pcDNA3.1(-)-Arl8b (Q75L)-HA	Full-length human Arl8b Q75L with C-terminal HA tagged cloned into the pcDNA3.1(-) vector	Described previously (Marwaha et al., 2017)
pcDNA3.1(-)-Arl8b (T34N)-HA	Full-length human Arl8b T34N with C-terminal HA tagged cloned into the pcDNA3.1(-) vector	Described previously (Marwaha et al., 2017)
pEGFP-C1-RILP	N-terminal GFP-tagged RILP cloned into the pEGFP-C1 vector	Described previously (Marwaha et al., 2017)
pEGFP-C1-TMEM55B	N-terminal GFP-tagged TMEM55B cloned into the pEGFP-C1 vector	This study
pEGFP-C1-Rab11	N-terminal GFP-tagged Rab11 cloned into the pEGFP-C1 vector	Gift from Prof. Steve Caplan
pCMV-Tag2B-JIP4	N-terminal FLAG-tagged human JIP4 (isoform 2) cloned into the pCMV-Tag2B vector	Gift from Dr. Clement Lee
pLJC5-TMEM192-3xHA	C-terminal HA-tagged TMEM192 cloned into the pLJC5 vector	Addgene plasmid # 102930
pLJC5-TMEM192-2xFLAG	C-terminal FLAG-tagged TMEM192 cloned into the pLJC5 vector	Addgene plasmid # 102929
Mito-Rab7-HA	Rab7A (QL)-BirA-HA-MAO	Addgene plasmid # 128904
Mito-Arl8b-HA	Human Arl8b (lacking first 17 aa) cloned into the Rab7A (QL)-BirA-HA-MAO vector by replacing Rab7 (QL) cassette	This study
Mito-FRB	Mito (Tom70p) fused to FRB	Gift from Prof. Martin Lowe
2x-FKBP-GFP	FKBP fused to GFP cloned into the pcDNA3.1(-) vector	This study
2x-FKBP-GFP-RUFY3	FKBP fused to GFP-RUFY3 cloned into the pcDNA3.1(-) vector	This study
Bacterial expression constructs:		
pGEX6P2-RUFY3	Human RUFY3 (1-620 aa) cloned into the pGEX6P2 vector	This study
pGEX6P2-RUFY3 (Δ446-561)	Human RUFY3 lacking amino acids 446-561 cloned into the pGEX6P2 vector	This study

pGEX6P2-RUFY3 (441-561)	Human RUFY3 amino acids 441-561 cloned into the pGEX6P2 vector	This study
pGEX6P2-RUFY3 RK→A (441-561)	Human RUFY3 (441-561 aa) with point mutations at amino acid positions R462 and K465 to A; cloned into the pGEX6P2 vector	This study
pETDuet-1-Arl8b	Full-length human Arl8b with N-terminal His tag cloned into the pETDuet-1 vector	This study
pGEX4T3-Arl8b	Full-length human Arl8b with N-terminal GST tag cloned into the pGEX4T3 vector	Described previously (Marwaha et al., 2017)

Supplementary Table 2: List of antibodies used in this study.

(WB: Western Blot; IF: Immunofluorescence; IP: Immunoprecipitation)

Antibody	Source	Identifier
Rabbit anti-RUFY3 (WB-1:1500)	Novus Biologicals	NBP1-89614
Rabbit anti-JIP4 (WB-1:1000; IF-1:100; IP-1 µg)	Cell Signaling Technology	5519
Rabbit anti-Rab5 (WB-1:1000)	Cell Signaling Technology	2143
Rabbit anti-Calreticulin (WB-1:1000)	Cell Signaling Technology	12238
Rabbit anti-Catalase (WB-1:1000)	Cell Signaling Technology	12980
Rabbit anti-Rab7 (WB-1:1000)	Cell Signaling Technology	9367
Rabbit anti-Arl8b (WB-1:1000; IF-1:30)	Cell Signaling Technology	56085
Rabbit anti-FLAG tag (IF-1:500)	Cell Signaling Technology	2368
Rabbit anti-LC3B (WB-1:1000)	Cell Signaling Technology	3868
Rabbit anti-LAMP1 (WB-1:5000; IF-1:1000)	Abcam	ab24170
Rabbit anti-Cathepsin D (WB-1:1500; IF-1:200)	Abcam	ab75852
Rabbit anti-TfR (IF-1:500)	Abcam	ab84036
Rabbit anti-RUFY3 (IP-1 µg)	Abcam	ab237511
Rabbit anti-VDAC (WB-1:1000)	Thermo Fisher Scientific	PA1-954A

Rabbit anti-FLAG tag (WB-1:4000)	Thermo Fisher Scientific	PA1-984B
Rabbit anti-LC3 (IF-1:1000)	MBL International Corporation	PM036
Rabbit anti-HA tag (WB-1:4000; IF-1:500)	Sigma-Aldrich	H6908
Mouse anti-HA-conjugated agarose beads (IP-12 µL slurry)	Sigma-Aldrich	A2095
Mouse IgG-conjugated agarose beads (IP-12 µL slurry)	Sigma-Aldrich	A0919
Rabbit IgG-conjugated agarose beads (IP-12 µL slurry)	Sigma-Aldrich	A2909
Rabbit anti-Arl8 (WB-1:1000)	Custom-made	Previously described (Garg S et al., Immunity 2011)
Mouse anti-Rab5 (IF-1:200)	BD Bioscience	610281
Mouse anti-LAMP1 (IF-1:500)	BD Bioscience	555798
Mouse anti-p150 (WB-1:1500; IF-1:100)	BD Bioscience	610474
Mouse anti-CD63 (IF-1:200)	BD Bioscience	556019
Mouse anti-DIC (WB-1:5000)	BioLegend	904901
Mouse anti-HA tag (WB-1:4000; IF-1:500)	BioLegend	901503
Anti-FLAG affinity gel (IP-12 µL slurry)	BioLegend	651503
Mouse anti-Tom-20 (IF-1:500)	Santa Cruz Biotechnology	sc-17764

Mouse anti-Arl8-conjugated-agarose beads (IP-30 µL slurry)	Santa Cruz Biotechnology	sc-398635 AC
Mouse anti-Rab7 (IF-1:30)	Santa Cruz Biotechnology	sc-376362
Mouse anti-GAPDH (WB-1:2000)	Santa Cruz Biotechnology	sc-166574
Mouse anti-β-tubulin (WB-1:4000)	Sigma-Aldrich	T4026
Mouse anti-FLAG tag (WB-1:4000; IF-1:500)	Sigma-Aldrich	F1804
Mouse anti-His tag (WB-1:5000)	Sigma-Aldrich	SAB1305538
Mouse anti-GST tag (WB-1:5000)	Thermo Fisher Scientific	MA4-004
Alexa-Fluor 488-conjugated goat anti-rabbit IgG (IF-1:500)	Thermo Fisher Scientific	A-11034
Alexa-Fluor 568-conjugated goat anti-rabbit IgG (IF-1:500)	Thermo Fisher Scientific	A-11036
Alexa-Fluor 488-conjugated goat anti-mouse IgG (IF-1:500)	Thermo Fisher Scientific	A-11029
Alexa-Fluor 568-conjugated goat anti-mouse IgG (IF-1:500)	Thermo Fisher Scientific	A-11031
Alexa-Fluor 633-conjugated goat anti-mouse IgG (IF-1:500)	Thermo Fisher Scientific	A-21050
HRP-conjugated goat anti-rabbit IgG (WB-1:5000)	Jackson ImmunoResearch	111-035-144
HRP-conjugated goat anti-mouse IgG (WB-1:5000)	Jackson ImmunoResearch	115-035-166
