# Supplementary material for: RUFY3 links Arl8b and JIP4-Dynein complex to regulate lysosome size and positioning
Source: Nat Commun. 2022 Mar 21;13:1540. doi: 10.1038/s41467-022-29077-y (PMC8938454; doi:10.1038/s41467-022-29077-y)
Supplement: Supplementary file 3 — Description of Additional Supplementary Files [file 41467_2022_29077_MOESM3_ESM.pdf]

Description of Additional Supplementary Files

File name: Supplementary Data 1

Description: List of RUFY3 interacting proteins isolated from HEK293T cell lysates by GST-pulldown assay and identification by mass spectrometry.

File name: Supplementary Movie 1

Description: Dynamics of lysotracker-labeled lysosomes in HeLa cells. Live-cell imaging of HeLa cells treated with control siRNA and incubated with Lysotracker (LTR DND-99) to label lysosomes. Time-lapse series were captured with frame time of 0.67 sec, and the movie is shown at 10 frames/sec. Scale Bar: 10 µm

File name: Supplementary Movie 2

Description: Effects of RUFY3 depletion on the dynamics of lysotrackerlabeled lysosomes. Live-cell imaging of HeLa cells treated with RUFY3 siRNA and incubated with Lysotracker (LTR DND-99) to label lysosomes. Time-lapse series were captured with frame time of 0.67 sec, and the movie is shown at 10 frames/sec. Scale Bar: 10 µm

File name: Supplementary Movie 3

Description: Effects of dynein depletion on the dynamics of lysotrackerlabeled lysosomes. Live-cell imaging of HeLa cells treated with DHC siRNA and incubated with Lysotracker (LTR DND-99) to label lysosomes. Time-lapse series were captured with frame time of 0.67 sec, and the movie is shown at 10 frames/sec. Scale Bar: 10 µm
