# Supplementary material for: RUFY3 links Arl8b and JIP4-Dynein complex to regulate lysosome size and positioning
Source: Nat Commun. 2022 Mar 21;13:1540. doi: 10.1038/s41467-022-29077-y (PMC8938454; doi:10.1038/s41467-022-29077-y)
Supplement: Supplementary file 8 — Reporting Summary [file 41467_2022_29077_MOESM8_ESM.pdf]

nature research

Corresponding author(s): Amit Tuli

Last updated by author(s): Feb 2, 2022

Reporting Summary

Nature Research wishes to improve the reproducibility of the work that we publish. This form provides structure for consistency and transparency in reporting. For further information on Nature Research policies, see our Editorial Policies and the Editorial Policy Checklist.

Statistics

For all statistical analyses, confirm that the following items are present in the figure legend, table legend, main text, or Methods section.

n/a	Confirmed	
☐	☒	The exact sample size (*n*) for each experimental group/condition, given as a discrete number and unit of measurement
☐	☒	A statement on whether measurements were taken from distinct samples or whether the same sample was measured repeatedly
☐	☒	The statistical test(s) used AND whether they are one- or two-sided<br>*Only common tests should be described solely by name; describe more complex techniques in the Methods section.*
☒	☐	A description of all covariates tested
☒	☐	A description of any assumptions or corrections, such as tests of normality and adjustment for multiple comparisons
☐	☒	A full description of the statistical parameters including central tendency (e.g. means) or other basic estimates (e.g. regression coefficient) AND variation (e.g. standard deviation) or associated estimates of uncertainty (e.g. confidence intervals)
☐	☒	For null hypothesis testing, the test statistic (e.g. F, t, r) with confidence intervals, effect sizes, degrees of freedom and P value noted<br>*Give P values as exact values whenever suitable.*
☒	☐	For Bayesian analysis, information on the choice of priors and Markov chain Monte Carlo settings
☒	☐	For hierarchical and complex designs, identification of the appropriate level for tests and full reporting of outcomes
☐	☒	Estimates of effect sizes (e.g. Cohen's d, Pearson's r), indicating how they were calculated

Our web collection on statistics for biologists contains articles on many of the points above.

Software and code

Policy information about availability of computer code

Data collection

ZEN 2012 v. 8.0.1.273 (Zeiss) for Confocal and Zen Black v. 3.0 SR (Zeiss) for SIM, Tecan infinite control 2.0.10.0 for pH Measurement and BD FACS Diva software v. 8.0.1 (BD Biosciences) for Flow cytometry.

Data analysis

Data analysis was done using Fiji v. 2.1.0 (NIH). For analysis of lysosome count and size from immunofluorescence micrographs, "analyze particle tool" function of Fiji software was used. For measurement of lysosome diameter from TEM images, "line tool" function of Fiji software was used. For measurement of cell surface area, "freehand" and "measure function" tools of Fiji software were used. To perform measurement of fractional distance, "plot profile", "freehand selection tool" and "clear outside function" tools of Fiji software were used.

For single-particle tracking, Trackmate v. 7.4.0. plugin was used.

For co-localization analysis, JACoP plugin was used.

For analysis of Flow cytometry based experiments, BD FlowJo v. 10.0.1. software was used.

Statistics and graphing was done using Prism v. 8.0 (GraphPad) and Excel 2013 (Microsoft).

All figures were assembled using Fiji v. 2.1.0 (NIH), Adobe Illustrator v. 24.3, Adobe Photoshop v. 22.0.0, and Imaris X64. 9.2.0 (BitPlane).

For manuscripts utilizing custom algorithms or software that are central to the research but not yet described in published literature, software must be made available to editors and reviewers. We strongly encourage code deposition in a community repository (e.g. GitHub). See the Nature Research guidelines for submitting code & software for further information.

Data

Policy information about availability of data

All manuscripts must include a data availability statement. This statement should provide the following information, where applicable:

- Accession codes, unique identifiers, or web links for publicly available datasets
- A list of figures that have associated raw data
- A description of any restrictions on data availability

Raw data files of mass spectrometry results were deposited onto ProteomeXchange consortium with identifier PXD027010 (https://www.ebi.ac.uk/pride/archive/projects/PXD027010). All relevant data supporting this study's findings are presented in the manuscript and supplementary information. Raw data, uncropped Western blots and yeast-two hybrid plates scan are available in the Source Data file which is provided with the manuscript.

Field-specific reporting

Please select the one below that is the best fit for your research. If you are not sure, read the appropriate sections before making your selection.

☒ Life sciences ☐ Behavioural & social sciences ☐ Ecological, evolutionary & environmental sciences

For a reference copy of the document with all sections, see nature.com/documents/nr-reporting-summary-flat.pdf

Life sciences study design

All studies must disclose on these points even when the disclosure is negative.

Sample size	All sample sizes are listed in detail in the figure legends of the relevant figures. Samples sizes were determined based on the estimates from preliminary experiments and similar studies (Willett R. et al., Nature Communications, 1580 (2017) and Marwaha R. et al., Journal of Cell Biology, 216 (2017)) so that reasonable statistical analysis could be conducted. A minimum of three biological replicates were taken for each experimental set, unless otherwise mentioned.
Data exclusions	Any transfected cells that were expressing excessively high levels of a protein that visibly altered cell morphology or exhibited saturated signal intensities were excluded from analysis. Further, binucleated cells and non-uniform shaped cells were excluded from analysis.
Replication	Experiments were conducted multiple times and were reproducible, as indicated in the legends to each figure. Some of the experiments were replicated in multiple cell lines.
Randomization	Randomization was not performed as all the cells were imaged and analyzed in the same way.
Blinding	Blinding was not performed as cells were prepared and analyzed by the same investigator.

Reporting for specific materials, systems and methods

We require information from authors about some types of materials, experimental systems and methods used in many studies. Here, indicate whether each material, system or method listed is relevant to your study. If you are not sure if a list item applies to your research, read the appropriate section before selecting a response.

Materials & experimental systems

n/a	Involved in the study
☐	☒ Antibodies
☐	☒ Eukaryotic cell lines
☒	☐ Palaeontology and archaeology
☒	☐ Animals and other organisms
☒	☐ Human research participants
☒	☐ Clinical data
☒	☐ Dual use research of concern

Methods

n/a	Involved in the study
☒	☐ ChIP-seq
☐	☒ Flow cytometry
☒	☐ MRI-based neuroimaging

Antibodies

Antibodies used	All the antibodies used in this study are listed in Supplementary Table 2.<br><br>Key: WB (Western blotting), IF (Immunofluorescence) and IP (Immunoprecipitation)<br><br>1) Rabbit anti-RUFY3 (WB-1:1500) Novus Biologicals NBP1-89614<br>2) Rabbit anti-JIP4 (WB-1:1000; IF-1:100; IP-1 µg) Cell Signaling Technology 5519<br>3) Rabbit anti-Rab5 (WB-1:1000) Cell Signaling Technology 2143

4) Rabbit anti-Calreticulin (WB-1:1000) Cell Signaling Technology 12238
5) Rabbit anti-Catalase (WB-1:1000) Cell Signaling Technology 12980
6) Rabbit anti-Rab7 (WB-1:1000) Cell Signaling Technology 9367
7) Rabbit anti-Arl8b (WB-1:1000; IF-1:30) Cell Signaling Technology 56085
8) Rabbit anti-FLAG (IF-1:500) Cell Signaling Technology 2368
9) Rabbit anti-LC3B (WB-1:1000) Cell Signaling Technology 3868
10) Rabbit anti-LAMP1 (WB-1:5000; IF-1:1000) Abcam ab24170
11) Rabbit anti-Cathepsin D (WB-1:1500; IF-1:200) Abcam ab75852
12) Rabbit anti-TfR (IF-1:500) Abcam ab84036
13) Rabbit anti-RUFY3 (IP-1 µg) Abcam ab237511
14) Rabbit anti-VDAC (WB-1:1000) Thermo Fisher Scientific PA1-954A
15) Rabbit anti-FLAG (WB-1:4000) Thermo Fisher Scientific PA1-984B
16) Rabbit anti-LC3 (IF-1:1000) MBL International Corporation PM036
17) Rabbit anti-HA (WB-1:4000; IF-1:500) Sigma-Aldrich H6908
18) Mouse anti-HA-conjugated agarose beads (IP-12 µL slurry) Sigma-Aldrich A2095
19) Mouse IgG-conjugated agarose beads (IP-12 µL slurry) Sigma-Aldrich A0919
20) Rabbit IgG-conjugated agarose beads (IP-12 µL slurry) Sigma-Aldrich A2909
21) Rabbit anti-Arl8 (WB-1:1000) Custom-made Previously described (Garg S et al., Immunity 2011)
22) Mouse anti-Rab5 (IF-1:200) BD Bioscience 610281
23) Mouse anti-LAMP1 (IF-1:500) BD Bioscience 555798
24) Mouse anti-p150 (WB-1:1500; IF-1:100) BD Bioscience 610474
25) Mouse anti-CD63 (IF-1:200) BD Bioscience 556019
26) Mouse anti-DIC (WB-1:5000) BioLegend 904901
27) Mouse anti-HA (WB-1:4000; IF-1:500) BioLegend 901503
28) Anti-FLAG affinity gel (IP-12 µL slurry) BioLegend 651503
29) Mouse anti-Tom-20 (IF-1:500) Santa Cruz Biotechnology sc-17764
30) Mouse anti-Arl8-conjugated-agarose beads (IP-30 µL slurry) Santa Cruz Biotechnology sc-398635 AC
31) Mouse anti-Rab7 (IF-1:30) Santa Cruz Biotechnology sc-376362
32) Mouse anti-GAPDH (WB-1:2000) Santa Cruz Biotechnology sc-166574
33) Mouse anti-β-tubulin (WB-1:4000) Sigma-Aldrich T4026
34) Mouse anti-FLAG (WB-1:4000; IF-1:500) Sigma-Aldrich F1804
35) Mouse anti-His (WB-1:5000) Sigma-Aldrich SAB1305538
36) Mouse anti-GST (WB-1:5000) Thermo Fisher Scientific MA4-004
37) Alexa-Fluor 488-conjugated goat anti-rabbit IgG (IF-1:500) Thermo Fisher Scientific A-11034
38) Alexa-Fluor 568-conjugated goat anti-rabbit IgG (IF-1:500) Thermo Fisher Scientific A-11036
39) Alexa-Fluor 488-conjugated goat anti-mouse IgG (IF-1:500) Thermo Fisher Scientific A-11029
40) Alexa-Fluor 568-conjugated goat anti-mouse IgG (IF-1:500) Thermo Fisher Scientific A-11031
41) Alexa-Fluor 633-conjugated goat anti-mouse IgG (IF-1:500) Thermo Fisher Scientific A-21050
42) HRP-conjugated goat anti-rabbit IgG (WB-1:5000) Jackson ImmunoResearch 111-035-144
43) HRP-conjugated goat anti-mouse IgG (WB-1:5000) Jackson ImmunoResearch 115-035-166

Validation

All antibodies used were selected based on supplier's recommendation for the particular application.

Key: WB (Western blotting), IF (Immunofluorescence) and IP (Immunoprecipitation)

1) Rabbit anti-RUFY3 antibody was validated by the supplier (Novus Biologicals) to work in WB. We have also validated this antibody in multiple human cell lines by the loss of recognition upon siRNA/shRNA against RUFY3.
2) Rabbit anti-JIP4 antibody was validated by the supplier (Cell Signaling Technology) to work in WB and IF. We have also tested this antibody by siRNA approach.
3) Rabbit anti-Rab5 antibody was validated by the supplier (Cell Signaling Technology) to work in WB in multiple cell lines. In our experiments, we have also observed band of expected molecular weight for Rab5.
4) Rabbit anti-Calreticulin antibody was validated by the supplier (Cell Signaling Technology) and other publication (DOI: 10.1111/cas.14976) to work in WB.
5) Rabbit anti-Catalase antibody was validated by the supplier (Cell Signaling Technology) and other publications (DOI: 10.1093/hmg/ddv489 and DOI: 10.1371/journal.pone.0168276) to work in WB.
6) Rabbit anti-Rab7 antibody was validated by the supplier (Cell Signaling Technology) and in our previous publication (DOI: 10.1083/jcb.201607085) to work in WB.
7) Rabbit anti-Arl8b antibody was validated by the supplier (Cell Signaling Technology) for WB in multiple cell lines. We have also tested this antibody in WB and IF in HeLa cells using siRNA approach.
8) Rabbit anti-FLAG tag antibody (Cell Signaling Technology) was previously shown to work in IF in multiple publications (DOI: 10.1371/journal.ppat.1005556; DOI: 10.1038/ncb3581 and DOI: 10.1158/0008-5472.CAN-14-0169).
9) Rabbit anti-LC3B antibody was validated by the supplier (Cell Signaling Technology) and in our previous publication (DOI: 10.1083/jcb.201607085) to work in WB.
10) Rabbit anti-LAMP1 antibody was validated by the supplier (Abcam) and in our previous publication (DOI: 10.1083/jcb.201607085) to work in WB and IF.
11) Rabbit anDOI: 10.1038/s41467-021-23715-7ti-Cathepsin D antibody was validated by the supplier (Abcam) and other publication (DOI: 10.1038/s41467-021-22389-5) to work in WB and IF.
12) Rabbit anti-TfR antibody was validated by the supplier (Abcam) and other publication (DOI: 10.3390/ijms22020498) to work in IF.
13) Rabbit anti-RUFY3 antibody (Abcam) was validated to work in IP in our lab. In our experiments, we observed band of expected molecular weight for RUFY3.
14) Rabbit anti-VDAC antibody was validated by the supplier (Thermo Fisher Scientific) to work in WB when tested in multiple human cell lines.
15) Rabbit anti-FLAG tag antibody (Thermo Fisher Scientific) was previously shown to work in WB in other publications (DOI: 10.1074/mcp.RA120.002370 and DOI: 10.1128/mBio.02410-20).
16) Rabbit anti-LC3 antibody was validated by the supplier (MBL International Corporation) and in other publication (DOI: 10.1038/nature07383) to work in IF.

17) Rabbit anti-HA tag antibody was extensively validated by the supplier (Sigma-Aldrich) to work in WB and IF.
18) Mouse anti-HA tag antibody-conjugated agarose bead was validated by the supplier (Sigma-Aldrich) and in our previous publication (DOI: 10.1083/jcb.201607085) to work in IP.
19) Mouse IgG antibody-conjugated agarose bead was validated by the supplier (Sigma-Aldrich) and previously shown in other publications (DOI: 10.7554/eLife.49677 and DOI: 10.1093/nar/gku479) to work in IP.
20) Rabbit IgG antibody-conjugated agarose bead was validated by the supplier (Sigma-Aldrich) and previously shown in other publications (DOI: 10.1016/j.molcel.2009.01.019 and DOI: 10.1371/journal.pone.0081583) to work in IP.
21) Rabbit anti-Arl8 antibody was previously validated in our publications (DOI: 10.4049/jimmunol.1700829; DOI: 10.1371/journal.ppat.1006700; DOI: 10.1083/jcb.201607085; DOI: 10.1091/mbc.E13-05-0259 and DOI: 10.1016/j.immuni.2011.06.009) to work in WB.
22) Mouse anti-Rab5 antibody was validated by the supplier (BD Bioscience) to work in IF in human cells.
23) Mouse anti-LAMP1 antibody was validated by the supplier (BD Bioscience) and previously shown in our publications (DOI: 10.4049/jimmunol.1700829; DOI: 10.1371/journal.ppat.1006700; DOI: 10.1083/jcb.201607085; DOI: 10.1091/mbc.E13-05-0259 and DOI: 10.1016/j.immuni.2011.06.009) to work in IF.
24) Mouse anti-p150 antibody was validated by the supplier (BD Bioscience) and previously shown in a publication (DOI: 10.1083/jcb.201804183) to work in WB and IF.
25) Mouse anti-CD63 antibody was validated by the supplier (BD Bioscience) to work in IF during development.
26) Mouse anti-DIC antibody was validated by the supplier (BioLegend) and previously shown in a publication (DOI: 10.1083/jcb.201804183) to work in WB.
27) Mouse anti-HA tag antibody was validated by the supplier (BioLegend) and previously shown in our publication (DOI: 10.1083/jcb.201607085) to work in WB and IF.
28) Anti-FLAG tag antibody affinity gel was validated by the supplier (BioLegend) and previously shown in other publications (DOI: 10.1084/jem.20200053 and DOI: 10.1016/j.molcel.2017.01.027) to work in IP.
29) Mouse anti-Tom-20 antibody was validated by the supplier (Santa Cruz Biotechnology) and previously shown in other publications (DOI: 10.1038/s41467-021-23715-7 and DOI: 10.1016/j.celrep.2021.108689) to work in IF.
30) Mouse anti-Arl8 antibody-conjugated-agarose bead (Santa Cruz Biotechnology) was validated in our lab to work in IP.
31) Mouse anti-Rab7 antibody was validated by the supplier (Santa Cruz Biotechnology) and previously shown in a publication (DOI: 10.3390/ijms23010486) to work in IF.
32) Mouse anti-GAPDH antibody was validated by the supplier (Santa Cruz Biotechnology) and previously shown in a publication (DOI: 10.1242/jcs.162651) to work in WB.
33) Mouse anti-β-tubulin antibody was validated by the supplier (Sigma-Aldrich) and previously shown in a publication (DOI: 10.1074/jbc.M110.194928) to work in WB.
34) Mouse anti-FLAG tag antibody was validated by the supplier (Sigma-Aldrich) and previously shown in our publication (DOI: 10.1083/jcb.201607085) to work in WB and IF.
35) Mouse anti-His tag antibody was validated by the supplier (Sigma-Aldrich) and previously shown in our publication (DOI: 10.1083/jcb.201607085) to work in WB.
36) Mouse anti-GST tag antibody was validated by the supplier (Thermo Fisher Scientific) and previously shown in a publication (DOI: 10.3389/fnmol.2020.00031) to work in WB.
37) Alexa-Fluor 488-conjugated goat anti-rabbit IgG antibody was extensively validated by the supplier (Thermo Fisher Scientific) for work in IF.
38) Alexa-Fluor 568-conjugated goat anti-rabbit IgG antibody was extensively validated by the supplier (Thermo Fisher Scientific) for work in IF.
39) Alexa-Fluor 488-conjugated goat anti-mouse IgG antibody was extensively validated by the supplier (Thermo Fisher Scientific) for work in IF.
40) Alexa-Fluor 568-conjugated goat anti-mouse IgG antibody was extensively validated by the supplier (Thermo Fisher Scientific) for work in IF.
41) Alexa-Fluor 633-conjugated goat anti-mouse IgG antibody was extensively validated by the supplier (Thermo Fisher Scientific) for work in IF.
42) HRP-conjugated goat anti-rabbit IgG antibody was extensively validated by the supplier (Jackson ImmunoResearch) for work in WB.
43) HRP-conjugated goat anti-mouse IgG antibody was extensively validated by the supplier (Jackson ImmunoResearch) for work in WB.

Eukaryotic cell lines

Policy information about cell lines

Cell line source(s)	HeLa, HEK293T, U2OS, A549 and ARPE-19 cells were obtained from American Type Culture Collection (ATCC) and expanded following their instructions.
Authentication	All cell lines used in this study were authenticated by ATCC and morphology was assessed by microscopy. Each cell type was cultured for no more than 15 passages.
Mycoplasma contamination	All cell lines used in this study were regularly screened for the absence of mycoplasma contamination by using the MycoAlert Mycoplasma Detection Kit (LT07-418, Lonza).
Commonly misidentified lines (See ICLAC register)	No misidentified cell lines were used in the study.

Flow Cytometry

Plots

Confirm that:

☒ The axis labels state the marker and fluorochrome used (e.g. CD4-FITC).

☒ The axis scales are clearly visible. Include numbers along axes only for bottom left plot of group (a 'group' is an analysis of identical markers).

☐ All plots are contour plots with outliers or pseudocolor plots.

☒ A numerical value for number of cells or percentage (with statistics) is provided.

Methodology

Sample preparation	To measure Lysotracker uptake, cells were incubated in phenol red-free complete DMEM media (Gibco) containing 100 nM Lysotracker Red (LTR-DND-99; Invitrogen) for 1 h at 37°C. Post-incubation period, media was removed, and cells were trypsinized, washed and resuspended in ice-cold 1XPBS and analyzed by flow cytometry. To measure the proteolytic activity of lysosome, cells were incubated in phenol red-free complete DMEM media (Gibco) containing 20 µg/mL BODIPY FL-BSA (BioVision) for 2 h at 37°C. Post-incubation period, media was removed, and cells were trypsinized, washed and resuspended in ice-cold 1XPBS and analyzed by flow cytometry. Sample acquisition was done with BD FACS Aria Fusion Cytometer using BD FACS Diva software version 8.0.1 (BD Biosciences). Data analysis was done using BD FlowJo version 10.0.1.
Instrument	BD FACS Aria Fusion Cytometer was used in the study.
Software	FACS Diva software version 8.0.1 (BD Biosciences)
Cell population abundance	30,000 cells per sample were analyzed.
Gating strategy	Viable cells were gated on a plot of FSC-A versus SSC-A.

☒ Tick this box to confirm that a figure exemplifying the gating strategy is provided in the Supplementary Information.
